# The prevalence of insomnia in the general population in China: A meta-analysis

**DOI:** 10.1371/journal.pone.0170772

**Published:** 2017-02-24

**Authors:** Xiao-Lan Cao, Shi-Bin Wang, Bao-Liang Zhong, Ling Zhang, Gabor S. Ungvari, Chee H. Ng, Lu Li, Helen F. K. Chiu, Grace K. I. Lok, Jian-Ping Lu, Fu-Jun Jia, Yu-Tao Xiang

**Affiliations:** 1 Shenzhen Key Laboratory for Psychological Healthcare & Shenzhen Institute of Mental Health, Shenzhen Kangning Hospital & Shenzhen Mental Health Center, Shenzhen, China; 2 Faculty of Mental health, Shenzhen University, Guangdong province, China; 3 Unit of Psychiatry, Faculty of Health Sciences, University of Macau, Macao SAR, China; 4 The Affiliated Mental Health Center, Tongji Medical College of Huazhong University of Science & Technology, Wuhan, China; 5 The National Clinical Research Center for Mental Disorders, China & Center of Depression, Beijing Institute for Brain Disorders & Mood Disorders Center, Beijing Anding Hospital, Capital Medical University, Beijing, China; 6 The University of Notre Dame Australia/Marian Centre, Perth, Australia; 7 School of Psychiatry & Clinical Neurosciences, University of Western Australia, Perth, Australia; 8 Department of Psychiatry, University of Melbourne, Melbourne, Victoria, Australia; 9 Department of Psychiatry, Chinese University of Hong Kong, Hong Kong SAR, China; 10 Kiang Wu Nursing College of Macau, Macao SRA, China; 11 Guangdong Mental Health Center, Guangdong General Hospital & Guangdong Academy of Medical Sciences, Guangdong Province, China; McMaster University, CANADA

## Abstract

This is the first meta-analysis of the pooled prevalence of insomnia in the general population of China. A systematic literature search was conducted via the following databases: PubMed, PsycINFO, EMBASE and Chinese databases (China National Knowledge Interne (CNKI), WanFang Data and SinoMed). Statistical analyses were performed using the Comprehensive Meta-Analysis program. A total of 17 studies with 115,988 participants met the inclusion criteria for the analysis. The pooled prevalence of insomnia in China was 15.0% (95% Confidence interval [CI]: 12.1%-18.5%). No significant difference was found in the prevalence between genders or across time period. The pooled prevalence of insomnia in population with a mean age of 43.7 years and older (11.6%; 95% CI: 7.5%-17.6%) was significantly lower than in those with a mean age younger than 43.7 years (20.4%; 95% CI: 14.2%-28.2%). The prevalence of insomnia was significantly affected by the type of assessment tools (Q = 14.1, P = 0.001). The general population prevalence of insomnia in China is lower than those reported in Western countries but similar to those in Asian countries. Younger Chinese adults appear to suffer from more insomnia than older adults.

**Trial Registration:** CRD 42016043620

## Introduction

Insomnia, which is characterized by difficulty initiating and maintaining sleep and/or waking up too early, appears to be one of the most frequent sleep complaints in the general population [1]. For example, approximately a third of the adult population in the USA suffer from insomnia [2]. It has been shown that insomnia has significant negative impact on daily functioning and is associated with work absenteeism, considerable impairment of quality of life, and increased medical and societal costs [3,4]. In addition, insomnia is frequently associated with a variety of psychiatric disorders, especially depression and anxiety [1,5,6].

Examining the prevalence of insomnia is essential for health professionals and policymakers to understand its influence on the general population and enact appropriate preventive strategies as well as to make reasonable health resource allocations and funding decisions based on the cost-burden to society. However, the prevalence of insomnia in the general population varies greatly across studies, ranging from 6% to 50% [7,8]. Such wide variation could be due to several factors, such as differences in the definition of insomnia, assessment tools and geographical locations. A systematic review of population studies on insomnia from Western countries interestingly found relatively low prevalence of insomnia (6%) using insomnia criteria according to Diagnostic and Statistical Manual of Mental Disorder-IV (DSM-IV) but high prevalence (30–48%) using insomnia symptoms [7]. Age and gender were the most commonly identified risk factors, with an increased prevalence in women and older adults [1].

To the best of our knowledge, no systematic review or meta-analysis of studies examining the prevalence of insomnia in China has been published. This study is a systematic, quantitative meta-analysis of the pooled prevalence of insomnia in the general adult population in China. In addition to the international literature, Chinese databases, which are not usually reviewed in the international literature, were also searched.

## Methods

### Inclusion and exclusion criteria

Studies fulfilling the following criteria were included: (a) cross-sectional studies examining prevalence of insomnia in the general population in mainland China (China thereafter); (b) available information on insomnia prevalence and sample size; (c) publications in full text either written in English or in Chinese. Studies conducted in specific subgroups (e.g., children, adolescents, and the elderly) or in special settings (e.g. hospitals, military) and studies used census sampling were excluded.

### Search strategy

A search was conducted for relevant papers for further detailed review in PubMed (S1 File), PsycINFO, EMBASE and Chinese databases (China National Knowledge Interne (CNKI), WanFang Data and SinoMed from database inception to April 10, 2016. Search words were listed as follows: (“insomnia” or “sleep problem” or “sleep disturbance” or “sleep disorder” or “sleep quality”) and (“prevalence” or “rate” or “epidemiology” or “survey” or “risk factor”) and (“China” or “Chinese”). Manual searches were also conducted by reviewing the reference lists from retrieved papers to find further relevant articles. Two reviewers independently screened the hits by reviewing titles and abstracts. The complete relevant articles were downloaded for further screening. If the same data were reported in more than one publication, only the paper with more complete data was included. Any disagreement was settled by discussion with the third author.

### Data extraction and quality assessment

Data extraction was independently performed by the two reviewers. A data extraction sheet included first author, year of publication, year of the study conducted, study setting, sample size, response rate, sampling method, measurement and criteria of insomnia, mean age, number and prevalence of insomnia for the whole sample and different demographic subgroups. To date, there have been no well-defined tools for assessing quality of observational epidemiological studies [9]. In line with other studies [10], sample size, sampling method and response rate were used as the criteria to assess the quality of included studies; i.e., those with larger sample size, random sampling and higher response rate were considered as having higher quality.

### Statistical analysis

All statistical analyses were conducted using the Comprehensive Meta-Analysis software, Version 2 (Biosta, Inc. USA). As the prevalence estimates of insomnia in most included studies were neither close to 50%, nor close to 0 or 100%, the logit transformation was used to form an unbounded estimate for analyses and the back transformation to proportion was performed for the final presentation [11,12]. The I^2^ statistic was used to evaluate heterogeneity of the studies. When the I^2^ statistic was greater than 50%, the random effect model was used for the meta-analysis [13], although being more conservative than the fixed effect model [14]. Forest plots were drawn to visualize the extent of heterogeneity across studies. Furthermore, the following 7 subgroup analyses were conducted in order to assess if any of them could reduce/explain the observed heterogeneity of the findings: (1) male vs. female; (2) mean age of study sample, using the median split of the mean age of study samples (≤43.7 yrs vs. >43.7 yrs); (3) survey year, using the median split of the survey years (≤2006 vs. >2006); (4) assessment tool: PSQI vs. AIS vs. standardized question; (5) sample size, using the median of sample size of included studies (≤5,358 vs. >5,358); (6) sampling method (convenience vs. random sampling); (7) response rate: ≥80% vs. <80%. Following other studies [15,16], in subgroup analyses some continuous variables (age, survey year and sample size) were dichotomized by a median split approach. Publication bias was estimated using Egger’s linear regression test and Funnel plots. All reported probabilities (p value) were two-sided, and p<0.05 was regarded statistically significant.

## Results

### Study characteristics

**Fig 1:**
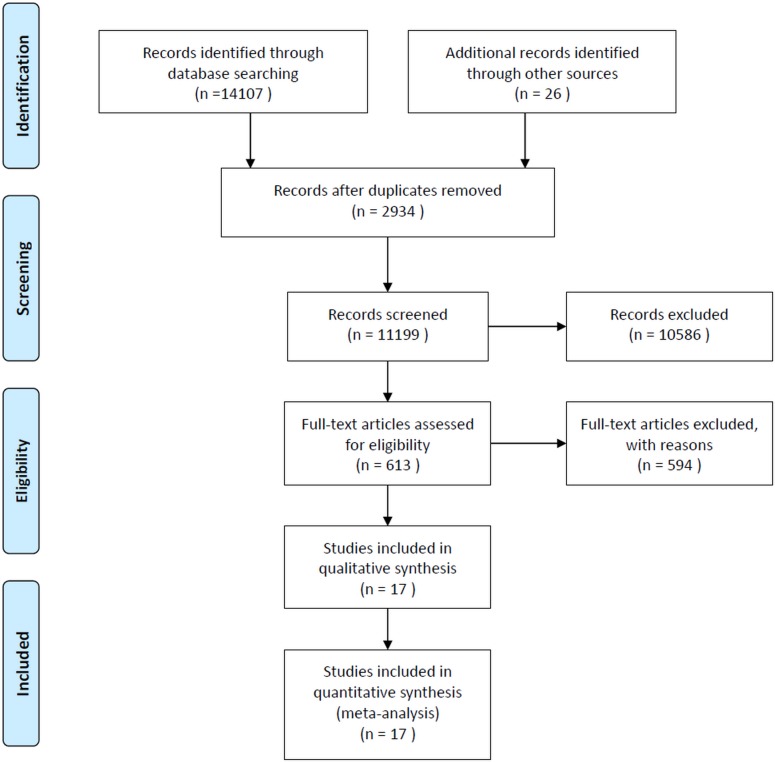
PRISMA flow diagram.

This meta-analysis was performed according to the Preferred Reporting Items for Systematic Reviews and Meta-Analyses (PRISMA) statement (S1 Table). A total of 11,199 eligible papers were identified by the initial literature search and 11,182 of them were excluded for various reasons (see Fig 1). Finally, 17 studies (2 in English and 15 in Chinese) met the inclusion criteria and were included for analyses [17–33]. Characteristics of the included studies are shown in Table 1. The studies were conducted between 1996 and 2011. Sample size ranged between 306 and 22,551, with a total number of 115,988 and a median of 5,385. The mean age of the study sample ranged from 28 to 49.4 years with the median age of 43.7 years. The Pittsburgh sleep quality index (PSQI) was the most commonly used tool to assess insomnia, followed by standardized questions designed by researchers, e.g., “Do you have insomnia during the last month” and Athens insomnia scale (ASI). Fourteen studies were conducted in northern or southern China [17–27,29,31,32], and only 3 studies [28,30,33] were in western China.

**Table 1:** Characteristics of studies included in the meta-analysis.

Authors and Publication year	Study site	Conducted year	Sampling method	Insomnia measures	Insomnia criteria	Response rate%	Mean age (range, yrs)	Sample size	Prevalence of insomnia %
Lu 2003	Shenzhen	1996	Multistage stratified random sampling	PSQI	PSQI>7	NR	43.3 (15–86)	948	10.8
Chen 2004	Xiamen	2004	Stratified random sampling	PSQI	PSQI>7	90.7	NR	2539	22.4
Li 2005	Gansu, Henan and Shandong province	2000	Multistage stratified random sampling	Do you have sleep disorder during past month?	Self-reported as yes	97.8	(≥18)	9777	6.9
Xiang 2008	Beijing	2003	Multistage, stratified, systematic, and probability sampling	yes-no questions about whether they had each of three classic forms of sleep disturbance specified in the DSM-IV	Lasting two weeks or longer in the past 12 months	94.8	(≥15)	5926	9.2
Su 2008	Hebei Province	2004	Multistage stratified random sampling	PSQI	PSQI>7	86.3	44 (18–95)	20716	11.6
Zhang 2008	Shandong Province	2004	Multistage stratified random sampling	PSQI	PSQI>7	94.0	(>18)	22551	13.18
Wen 2010	Xiamen	2010	Convenience sampling	PSQI	PSQI>7	88.3	33.5 (8–81)	497	29.38
Xie 2010	Henan Province	2006	Stratified random sampling	PSQI	PSQI≥7	99.1	28 (14–84)	1500	21.3
Dai 2011	Rizhao	2010	Stratified random sampling	AIS	AIS>6	96.4	45.2 (18–96)	9732	21.66
Liu 2011	Beijing	2010	Stratified random sampling	PSQI	PSQI>7	95.6	(22–82)	306	40.5
Sun 2011	Shanghai	NR	Stratified sampling	Do you have difficulty in falling sleep?	≥3 times/week and last at least 1 month or above	NR	(21–80)	980	32.86
Xu 2011	Bozhou, Xinjiang,	2009	Stratified random sampling	AIS	AIS>6	89.2	38.4 (18–79)	803	23.9
Ye 2014	Fujian Province	2009	Multistage stratified random sampling	PSQI	PSQI>7	NR	46.8±12.6(18–80)	5358	4.5
You 2014	Chongqing	NR	Multistage stratified random sampling	PSQI	PSQI>7	94.82	<18 - ≥60	1429	15.89
Gu 2015	Tianjin	2011	Multistage stratified random sampling	PSQI	PSQI>7	75.30	≥18	11618	6.6
Zhang 2015	Yibin, Sichuan,	NR	Multistage stratified random sampling	Do you have insomnia during the last month?	≥12 d in the past month	93.60	49.4±15.2 (18–98)	11227	14.9
Zhan 2016	Beijing	2007	Multistage stratified random sampling	During the last month, have you had insomnia?	≥3 times/week)	83.50	≥18	10054	8.7

AIS: Athens insomnia scale; NR: not report; PSQI: Pittsburgh sleep quality index

### Evaluation quality of the studies

Sixteen studies used stratified random sampling and only one used convenience sampling. While thirteen studies reported high response rates (≥80%), one study had a response rate of 75.3% and three did not report any response rate. Eight of the studies had a sample size larger than the median of 5,358 participants.

### Heterogeneity of studies and summary of insomnia prevalence

**Fig 2:**
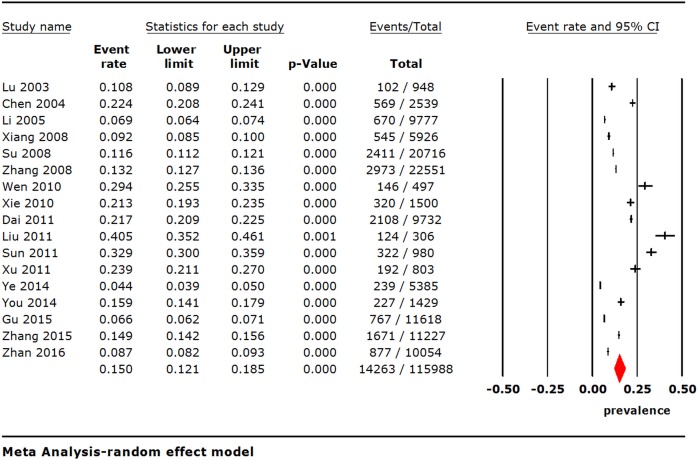
Point prevalence of insomnia in Chinese general population.

Using the random effects model, the pooled point prevalence of insomnia with a total sample size of 115,988 in Chinese general population was 15.0% (95% Confidence interval [CI], 12.1%-18.5%; I^2^ = 99.5%) (Fig 2).

### Publication bias

**Fig 3:**
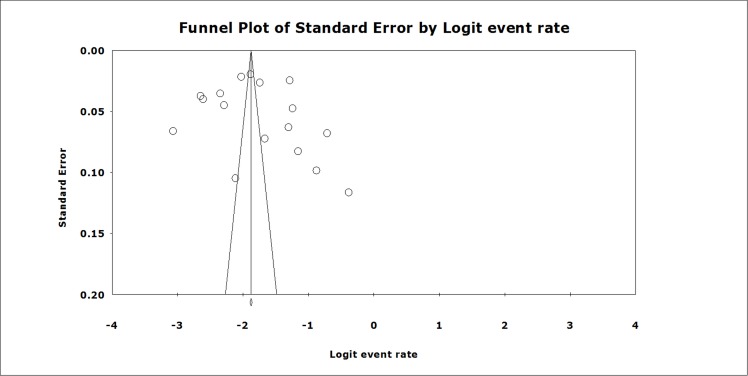
Funnel plot of publication bias for the included studies.

As Fig 3 shows, the funnel plots seem to be symmetrical as confirmed by Egger’s linear regression test (t = 0.54, p = 0.60), indicating that there is no publication bias.

### Subgroup analyses

There were no significant associations between prevalence of insomnia and gender and the year in which the survey was done. The median of mean age of participants in the studies was 43.7 years. The pooled prevalence of insomnia in the older subject group was 11.6% (95% CI: 7.5%-17.6%), which was significantly lower than the figure (20.4%; 95% CI: 14.2%-28.2%) in the younger subject group. In ten studies which used the PSQI to assess insomnia, their pooled prevalence of insomnia was 15.1% (95% CI: 11.4%-19.6%). From the five studies that assessed insomnia with standardized questions, e.g., “Did you have insomnia in the past month”, the pooled prevalence of insomnia was 12.5% (95% CI: 8.1%-18.9%). Only two studies used the AIS to assess insomnia which resulted in a pooled prevalence of 22.3% (95% CI: 20.4%-24.4%). There was significant difference in the pooled prevalence of insomnia between the above three groups (Q = 14.1, P = 0.001) (Table 2).

**Table 2:** Prevalence of insomnia according to socio-demographic characteristics.

Subgroups	Number of studies	Sample size	Sample size	Prevalence (%)	95% CI	I^2^ (%)	Q-value (p-value)
Male	14	45930	5046	14.2	11.1–18.0	98.8	2.84 (0.092)
Female	14	53396	7934	18.5	15.2–22.4	98.9	
Mean age of study samples ≤43.7 yrs	4	3748	760	20.4	14.2–28.2	96.3	4.0 (0.046)
Mean age of study samples >43.7 yrs	4	47060	6429	11.6	7.5–17.6	99.6	
Survey year≤2006	7	63957	7590	12.7	9.9–16.2	99.1	0.47(0.49)
Survey year>2006	7	38395	4453	15.6	9.1–25.6	99.7	
Assessment tool							
PSQI	10	67489	7878	15.1	11.4–19.6	99.3	14.1 (0.001)
AIS	2	10535	2300	22.3	20.4–24.4	54.5	
Standardized questions	5	37964	4085	12.5	8.1–18.9	99.5	
Sample size≤5358	9	14387	2241	19.9	12.9–29.3	99.1	5.48 (0.019)
Sample size>5358	8	101601	12022	10.9	8.3–14.1	99.6	
Convenience sampling	1	15349	3288	29.4	25.5–33.5	0	31.2 (<0.001)
Random sampling	16	115491	14117	14.4	11.5–17.8	99.5	
Response rate≥80%	13	11618	767	6.6	6.2–7.1	0	64.4 (<0.001)
Response rate <80%	1	97057	12833	16.6	13.5–20.3	99.3	

AIS: Athens insomnia scale; PSQI: Pittsburgh sleep quality index

In this meta-analysis, the quality of studies was assessed by sample size, sampling method and response rate. In order to examine the effects of the study quality on the results, further subgroup analyses were performed. The subgroups of “sample size ≤5,358 participants”, “convenience sampling” and “response rate <80%” were significantly associated with higher prevalence of insomnia (p<0.05) (Table 2).

## Discussion

This was the first meta-analysis of the general population in China examining the prevalence of insomnia. Most studies assessed the prevalence of insomnia symptoms using the PSQI or AIS, while five studies used standardized questions “yes-no” answers without any restrictive criteria or their frequency. No studies used insomnia diagnoses according to DSM-IV. The pooled prevalence of insomnia in China was 15.0%, which was lower than those in many Western countries (e.g., 37.2% in France and Italy, 27.1% in USA and 50.5% in Poland) [2,8], but similar to findings reported from other Asian countries (e.g., 15.3% in Japan and 17.3% in Singapore) [7]. However, the results of studies should be viewed with caution due to the confounding effects caused by discrepancy in sample sizes, response rates, sampling methods and measures on insomnia. In addition, no publication bias was found in this study which may be related to the nature of prevalence studies. Unlike meta-analysis comparing two treatments where negative results are often not published, prevalence studies in the general population are less likely to be subjected to publication bias especially if the sample size is adequate.

No gender difference in the prevalence of insomnia was observed in our study, which was inconsistent with other studies [34–36]. There was also no significant difference pre- or post-2006 in the prevalence of insomnia, indicating a lack of change over time. This was not consistent with other cross-sectional studies, which showed a steady increase in the prevalence of insomnia in the general population over time [37–39].

Many studies consistently reported an increased prevalence of insomnia with age [1,7]. This study found that the pooled prevalence of insomnia in younger subjects was significantly higher than in older subjects, which clearly indicates that young people are more likely to suffer insomnia. Due to the rapid urbanization and industrialization younger adults often face career stress and work long hours frequently at night, which disrupts their biological sleep rhythm and lead to insomnia [40]. In addition, the widespread use of new media, such as computers and smart phones in younger adults in China, may be associated with an increasing risk of insomnia. Also, in a meta-analysis the potential impact of ecological bias on the results could not be excluded [41,42].

The PSQI was the most common tool used to measure insomnia, followed by standardized questions and the AIS. The pooled prevalence of insomnia differed significantly between different assessment tools, thus supporting the notion that the prevalence could be largely determined by measurement tools [7]. Not surprisingly, the pooled prevalence in high quality epidemiology studies (larger sample size, random sampling and higher response rate) was lower than that of low quality studies, suggesting that well-designed studies are needed to accurately evaluate the prevalence of insomnia in future. The studies with lower response rate reported relatively higher prevalence of insomnia, which suggests that non-responders were less likely to have insomnia leading to selection bias to an uncertain degree. In addition, the prevalence of insomnia was relatively higher in studies with smaller sample size. While there is no known external factor that could cause a systemic distortion in smaller studies, we can only assume that the results of smaller studies may be relatively more unstable. This observation however requires further clarification.

There are several limitations to this meta-analysis. First, none of the studies used the DSM-IV insomnia diagnoses. Second, large heterogeneity was still present in the subgroup analyses, although heterogeneity is difficult to avoid in large meta-analysis of epidemiological surveys [43,44]. Third, some important information, such as comorbid psychiatric disorders, substance use and dependence, marital status and education level, were not available or incomplete in the publications, therefore could not be included in the analyses. Fourth, different definitions of insomnia were used in the studies, which made it difficult to interpret the results. However, this is a common challenge many studies face in the field of sleep medicine. In addition, the discrepancy in study characteristics, such as study sizes, response rates and sampling methods, made direct comparisons between studies difficult. Finally, although the logit transformation to pool the prevalence was performed as default in the Comprehensive Meta-Analysis software, potential variance stability may potentially exist in such transformation [12].

## Conclusions

This was the first meta-analysis of the pooled prevalence of insomnia in the general population of China. The pooled prevalence of insomnia is lower than those reported in Western countries, but similar to those in other Asian countries. Younger adults are more likely to suffer from insomnia than older adults.

## Supporting information

S1 Table PRISMA 2009 Checklist. (DOC)

S1 File Supplemental material-search terms in PubMed. (DOCX)
