# Supplementary material for: The prevalence of insomnia in the general population in China: A meta-analysis
Source: PLoS One. 2017 Feb 24;12(2):e0170772. doi: 10.1371/journal.pone.0170772 (PMC5325204; doi:10.1371/journal.pone.0170772)
Supplement: S1 File — (DOCX) [file pone.0170772.s002.docx]

SUPPLEMENTAL MATERIAL FOR ONLINE ONLY

| **Steps** | **Search term** | **Search string** |
| --- | --- | --- |
| 1 | insomnia | "Sleep Initiation and Maintenance Disorders"[Mesh] OR "insomnia"[All Fields] |
| 2 | sleep problem | "dyssomnias"[Mesh] OR "dyssomnias"[All Fields] OR "sleep problem"[All Fields] |
| 3 | sleep disturbance | "sleep disturbance"[All Fields] |
| 4 | sleep disorder | "sleep wake disorders"[MeSH Terms] OR "sleep wake disorders"[All Fields] OR "sleep disorder"[All Fields] |
| 5 | sleep quality | "sleep quality"[All Fields] |
| 6 | 1 OR 2 OR 3 OR 4 OR 5 |  |
| 7 | prevalence | "prevalence"[MeSH Terms] OR "prevalence"[All Fields] |
| 8 | rate | "rate"[All Fields] |
| 9 | epidemiology | "epidemiology"[Subheading] OR "epidemiology"[All Fields] OR "epidemiology"[MeSH Terms] |
| 10 | survey | "surveys and questionnaires"[MeSH Terms] OR ("surveys"[All Fields] AND "questionnaires"[All Fields]) OR "surveys and questionnaires"[All Fields] OR "survey"[All Fields] |
| 11 | risk factor | "risk factors"[MeSH Terms] OR "risk factors"[All Fields] |
| 12 | 7 OR 8 OR 9 OR 10 OR 11 |  |
| 13 | China | "china"[MeSH Terms] OR "china"[All Fields] |
| 14 | Chinese | "chinese"[All Fields] |
| 15 | 13 OR 14 |  |
| 16 | 6 and 12 and 15 |  |

Appendix S1: Search terms in PubMed.
